# Short Chain Fatty Acids Enhance Expression and Activity of the Umami Taste Receptor in Enteroendocrine Cells via a Gα_i/o_ Pathway

**DOI:** 10.3389/fnut.2020.568991

**Published:** 2020-10-29

**Authors:** Matilda Shackley, Yue Ma, Edward W. Tate, Alastair J. H. Brown, Gary Frost, Aylin C. Hanyaloglu

**Affiliations:** ^1^Section of Nutrition Research, Department of Metabolism, Digestion, and Reproduction, Imperial College London, London, United Kingdom; ^2^Institute of Reproductive and Developmental Biology, Department of Metabolism, Digestion, and Reproduction, Imperial College London, London, United Kingdom; ^3^Department of Chemistry, Imperial College London, London, United Kingdom; ^4^Translational Sciences, Sosei Heptares, Cambridge, United Kingdom

**Keywords:** short chain fatty acid (SCFA), enteroendocrine cell, umami taste receptor, GPCR (G protein coupled receptor), heterotrimeric G protein

## Abstract

The short chain fatty acids (SCFAs) acetate, butyrate and propionate, are produced by fermentation of non-digestible carbohydrates by the gut microbiota and regulate appetite, adiposity, metabolism, glycemic control, and immunity. SCFAs act at two distinct G protein coupled receptors (GPCRs), FFAR2 and FFAR3 and are expressed in intestinal enteroendocrine cells (EECs), where they mediate anorectic gut hormone release. EECs also express other GPCRs that act as nutrient sensors, thus SCFAs may elicit some of their health-promoting effects by altering GPCR expression in EECs and enhance gut sensitivity to dietary molecules. Here, we identify that exposure of the murine EEC STC-1 cell line or intestinal organoids to physiological concentrations of SCFAs enhances mRNA levels of the umami taste receptors TASR1 and TASR3, without altering levels of the SCFA GPCRs, FFAR2 and FFAR3. Treatment of EECs with propionate or butyrate, but not acetate, increased levels of umami receptor transcripts, while propionate also reduced CCK expression. This was reversed by inhibiting Gαi/o signaling with pertussis toxin, suggesting that SCFAs act through FFAR2/3 to alter gene expression. Surprisingly, neither a FFAR3 nor a FFAR2 selective ligand could increase TASR1/TASR3 mRNA levels. We assessed the functional impact of increased TASR1/TASR3 expression using unique pharmacological properties of the umami taste receptor; namely, the potentiation of signaling by inosine monophosphate. Activation of umami taste receptor induced inositol-1-phosphate and calcium signaling, and butyrate pretreatment significantly enhanced such signaling. Our study reveals that SCFAs may contribute to EEC adaptation and alter EEC sensitivity to bioactive nutrients.

## Introduction

After ingestion, physical, and chemical processes digest food into a large and dynamic array of metabolites within the gastrointestinal (GI) tract. The detection of these, via “nutrient sensing” mechanisms, results in the secretion of over twenty different peptides from enteroendocrine cells (EECs) (1). Of particular note are colonic short-chain fatty acids (SCFAs), the anaerobic fermentation of non-digestible carbohydrates, components of high-fiber diets. These are carboxylic acids with fewer than six carbons (Cs), which can reach high luminal concentrations of 10^−1^ M (2, 3). Ninety-five percent of the SCFAs produced in the GI tract are acetate (2Cs), propionate (3Cs) and butyrate (4Cs) (3, 4). These SCFAs, in particular propionate, are currently of interest, not only because of their ability to regulate anorectic gut hormone release, but also to promote weight loss, reduce abdominal adiposity and improve insulin sensitivity (5–8).

A large range of luminally expressed cell surface proteins is responsible for nutrient sensing. A significant proportion of these are members of the superfamily of G protein-coupled receptors (GPCRs) (8). SCFAs activate two distinct GPCRs that are known to be expressed in EECs, FFAR2 and FFAR3 (8–13). When expressed in heterologous cells, these two receptors display differential potency for each SCFA, which also differs between human and mouse receptor orthologs, yet propionate is the most potent of the SCFAs at both murine receptors (13–15). FFAR2 and FFAR3 both activate Gα_i/o_ signaling, and FFAR2 can also signal via Gα_q/11_ to release calcium (Ca^2+^) from intracellular stores; a pathway associated with its role in inducing gut hormone secretion from human and mouse EECs (5, 7, 8, 16–18). However, beyond regulating levels of gut hormone expression (19) and secretion (5, 7, 17, 18), our understanding of the additional roles of FFAR2/3 in EECs is limited.

A variety of GPCRs act as nutrient sensors in EECs, each responding to a distinct range of macromolecules and metabolites (8). As the GPCR expression in EECs is not static (20), one possibility is that nutrients can alter GPCR expression levels, adapting the sensitivity of the gut to other dietary molecules. There is evidence to support this; obese individuals have significantly different expression profiles of nutrient sensing GPCRs in their GI tract compared with lean controls, with significant gene expression changes in genes encoding GPCRs, such as umami taste receptor subunit TAS1R3 and long chain fatty acid receptor FFAR4 (21, 22). Further studies have demonstrated that there are significant differences in the mRNA expression of long and short chain fatty acid GPCRs and gustatory receptors in obese mice compared with lean controls (23), which are altered significantly following gastric bypass surgery (20). Overall, this suggests plasticity in the expression of nutrient sensing receptors, enabling dynamic adaptation to environmental factors. It is unknown whether the recently reported health benefits of increased colonic concentrations of SCFAs, such as propionate (5, 7, 17), are partly mediated by an underlying mechanism that alters the ability of the gut to sense other dietary components/metabolites.

In this study, we demonstrate that exposure of EECs to SCFAs can increase the gene expression of a specific gustatory GPCR, the umami taste receptor, without altering the levels of SCFA receptors. This altered gene expression was mediated by propionate and butyrate via a Gα_i/o_ signaling pathway, supporting a SCFA-GPCR signaling mechanism; however, synthetic FFAR2- or FFAR3-selective ligands could not mimic this. The increased expression of umami taste receptor subunits by SCFAs resulted in enhanced signaling from this receptor.

## Materials and Methods

### Cell Culture

STC-1 cells originate from enteroendocrine tumors in the duodenum of double transgenic mice (24). This cell line was used for all experiments, unless otherwise specified. STC-1 cells were cultured (95% O_2_; 5% CO_2_; 37°C) in Dulbecco's modified Eagle's Medium (DMEM) containing 4.5 g/L D-glucose, 4 mM L-Glutamine (Sigma), supplemented with 10% FBS (Sigma), 100 U/mL penicillin and 100 mg/L streptomycin (ThermoFisher; DMEM+/+).

### Intestinal Organoids

Ileum crypts were isolated with 12.5 mM EDTA from C57BL/6 mice and were embedded in Cultrex Reduced Growth Factor Basement Membrane Extract (BME) (R&D Systems, Abingdon, UK), diluted 1:1 in Complete Growth Medium (CGM), and seeded in a 48-well plate. CGM contained Advanced DMEM/F12 supplemented with 100 μg/ml Primocin, 10 mM HEPES, 1x Glutamax, 1X N2, 1X B27, 50 ng/ml murine EGF, 100 ng/ml murine Noggin, 1.25 mM N-acetylcysteine, 3 μM CHIR99021, and 10% R-spondin-1 conditioned medium. BME was polymerized for 30 min at 37°C, and 300 μl of CGM was added. CHIR99021 was removed from CGM 3 days after seeding, and medium was replaced every 2–3 days. Organoids were passaged every 7 days using Gentle Dissociation Reagent.

### Ligand Treatment

STC-1 cells were grown to 70–80% confluency before treatment with SCFAs. All SCFAs were stored in solid salt form (Sigma). Solutions (100 mM) were made fresh for every experiment by dissolving in DMEM+/+ for incubations ≥5 h and in serum-free media for incubations <5 h. 2-(4-chlorophenyl)-3-methyl-N-(thiazole-2-yl)butanamide (4-CMTB; Tocris) was used as a FFAR2-specific agonist and AR420626 (Cayman) was used as an FFAR-specific agonist, both at a working concentration of 10 μM.

### Quantitative-PCR

After incubations with SCFAs, TRIzol® Reagent (Life Technologies) was used to extract RNA from STC-1 cells or organoid cultures. After purification, 1 μg of each RNA sample was treated with an RNAse inhibitor (ThermoFisher), and a DNase I treatment kit (Life Technologies). SuperScript IV Reverse Transcriptase kit (Life Technologies) was used to synthesize complimentary DNA (cDNA). qPCR was performed using SYBR-Green PCR Mastermix kit (ThermoFisher). Each reaction was run in triplicates and cDNA was replaced with nuclease-free water as a negative control. Reactions were performed using the ABI StepONE sequence system. The 2^−ΔΔ*CT*^ method (25) was used for analysis of raw C_t_ values. Briefly, gene expression was normalized to the housekeeping gene β-actin, and values from treated cells were compared with the expression of untreated controls. All primer sequences used were purchased predesigned from Sigma Aldrich UK (sequences found in Supplementary Table 1). Serial dilution curves were performed to ensure primer efficacy of 90–110%.

### Measurement of Intracellular cAMP

All cAMP assays were performed in serum-free DMEM (Sigma) supplemented with 3-isobutyl-1-methylxanthine (IBMX; 0.5 mM; Sigma) to inhibit cAMP degradation by phosphodiesterases. cAMP concentrations were measured from cell lysates after cells were incubated for 5 min with synthetic agonists (10 μM) for FFAR2 (4-CMTB) or FFAR3 (AR420626) using the HTRF cAMP Dynamic 2 immunoassay kit (CisBio). Fluorescence was measured with a PHERAstar FSX plate reader (BMG Labtech) equipped with HTRF 337 optic module, with excitation at 340 nm and measurements of emission at 620 and 665 nm. cAMP levels were interpolated from an cAMP standard curve and normalized to protein concentration. All experiments were conducted in triplicate and repeated at least 3 times.

### Measurement of Intracellular Inosine-1-Phosphate (IP_1_)

IP_1_ signaling assays were performed after incubation with SCFAs to evaluate the response of STC-1 cells to L-monosodium glutamate (L-MSG; Sigma) and L-Alanine (L-Ala; Sigma), selected owing to their potency at the rodent umami taste receptor (26, 27). All reactions were performed in the presence and absence of inosine monophosphate (IMP, 2 mM) in serum-free DMEM (Sigma) supplemented with 50 mM LiCl (Sigma). After cells were treated with ligands (30 min), IP_1_ concentrations were measured from cell lysates using the HTRF IP-One immunoassay kit (CisBio). Fluorescence was measured and IP_1_ levels were quantified using the same methodology as the cAMP assay.

### Ca^2+^ Mobilization

Intracellular levels of Ca^2+^ were measured using the Fluo-4AM Direct Calcium Assay Kit (Invitrogen). STC-1 cells were incubated with a 1:1 ratio of opti-MEM media (Sigma, UK) to calcium dye Fluo-4-AM Direct for 30 min at 37°C and for a further 30 min at room temperature. Cells were imaged using a Leica Confocal Microscope (20X dry objective; 488 nm excitation). Movies were recorded at 1 fps for 60 s before addition of IMP/control (2 mM). After ensuring no Ca^2+^ mobilization in response to IMP, ligands (L-Ala or L-MSG) were added and movies were recorded until the readout returned to basal levels. All conditions for each experiment were conducted in duplicate and repeated at least three times. The fluorescence intensity of each cell was quantified using the ImageJ plugin Time Series Analyzer. The maximal intensity was obtained from subtracting the average background intensity (recorded before ligand addition) for each cell and averaged across 20 cells per condition.

### Statistical Analysis

Data are represented as the mean ± the standard error (SE) of results collected across at least three distinct experiments. GraphPad Prism was used to determine significance (*p* < 0.05), using unpaired Student's *t*-tests, One-way ANOVA with a Dunnett *post-hoc*, or Two-way ANOVA followed by a Bonferroni *post-hoc* test.

## Results

### A Physiologically Relevant Concentration of SCFAs Alters Expression of Taste Receptor and Gut Hormone Transcripts

**Figure 1:**
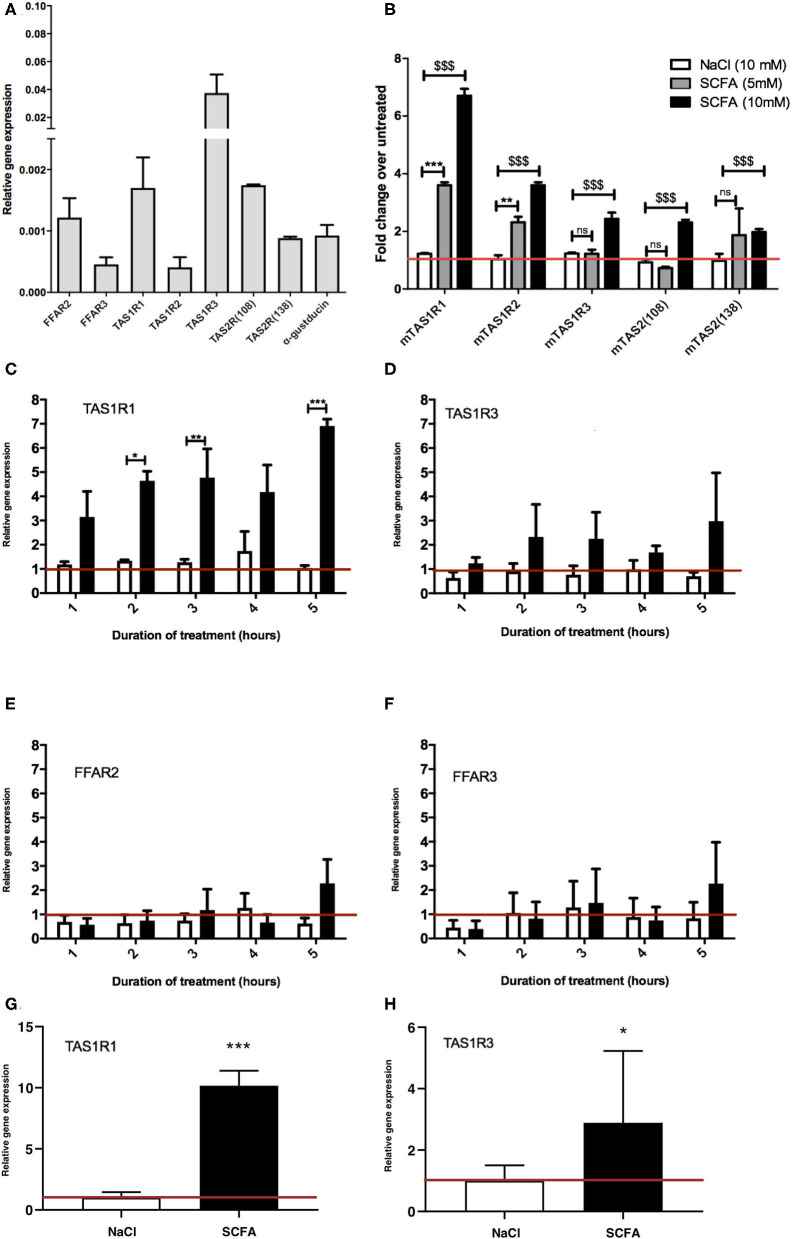
Exposure to SCFAs significantly changes the expression profile of taste receptors in STC-1 cells. **(A)** RNA was extracted from STC-1 cells for qPCR analysis of taste receptors TAS1R1, TAS1R3, TAS1R2, TAS2 (108), and TAS2 (138); free fatty acids receptors FFAR2 and FFAR3; and taste-specific G-protein α-gustducin, and normalized to the levels of housekeeping gene β-actin. **(B–G)** STC-1 cells were treated with NaCl (control; white bars), 5 mM SCFAs (gray bars), or 10 mM SCFA (black bars) for 2 h for (**B** only) and between 1 and 5 h as indicated, after which RNA was extracted, purified and quantified with qPCR. **(G,H)** Ileal intesinal organoids were treated with 10 mM NaCl or SCFAs for 5 h and RNA extracted and quantified via qPCR. Results are expressed as a fold change in expression over the untreated control and all data represent the average ± SEM, *n* = 3. The red line indicates a fold change of 1. **(A–F)** Two-way ANOVA, with Bonferroni *post hoc*, $$$*p* < 0.001 SCFA (10 mM) vs. NaCl control, **p < 0.01, ***p < 0.001 SCFA (5 mM) vs. NaCl control. **(G,H)** Unpaired *t*-test, *p < 0.05, ***p < 0.001.

A key aim of our study was to determine whether SCFA treatment of EECs would alter GPCRs previously demonstrated to be differentially expressed between obese and lean mice and humans (20–23), with a specific focus on the taste receptor GPCRs. Initially we confirmed that STC-1 cells expressed FFAR2, FFAR3, TAS1R1, TASR2, TAS1R3, the taste receptor-associated G-protein, α-gustducin, and two bitter taste receptors, TAS2R (108) and TAS2R (138), that were selected based on their potential involvement in bitter compound-induced Ca^2+^ signaling (28). We detected transcripts for all these genes in STC-1 cells, albeit in varying amounts (Figure 1A), confirming this cell-line represented an appropriate model in which to study potential interactions between SCFA signaling and the gustatory signaling system.

To determine whether SCFAs can influence the expression of taste GPCRs, STC-1 cells were incubated for 2 h with SCFAs in a 3:1:1 molar ratio of acetate:propionate:butyrate at 5 or 10 mM (final concentration) chosen to reflect the physiological SCFA concentrations in the proximal and distal colon (3, 4). qPCR was used to analyze the relative changes in expression of the transcripts of TAS1R1, TAS1R2, TAS1R2, TAS2 (108), and TAS2 (138). Incubation with 10 mM SCFAs significantly upregulated all taste receptors (*p* < 0.001 vs. control), whereas incubation at 5 mM only significantly upregulated TAS1R1 and TAS1R2 (Figure 1B). The largest fold change was observed with transcripts for TAS1R1 where SCFAs (10 mM) induced a 6.7-fold increase over basal levels (Figure 1B). Based on these initial observations we decided to investigate the mechanism of upregulation of the TAS1R1 subunit further. As TAS1R1 is only functionally active when it is heterodimerized with TAS1R3 (forming the umami taste receptor) (26, 27), we extended our investigation to include the TAS1R3 subunit. Treatment of cells with SCFAs (5 mM) over time (1–5 h) revealed that TAS1R1 was significantly upregulated following 2, 3, and 5 h of SCFA incubation (Figure 1C). Conversely, SCFAs at 5 mM did not affect the levels of TAS1R3 (Figures 1D,B). SCFAs did not alter the expression of SCFA receptors FFAR2 and FFAR3 (Figures 1E,F) at any time-point. The SCFA-dependent increases in TAS1R1 and TAS1R3 were also observed in 3D organoid cultures derived from small intestinal ileal crypts. Treatment of intestinal organoids with 10 mM SCFAs for 5 h resulted in a significant increase in umami taste receptor expression levels (Figure 1G,H).

The anorectic gut hormones glucagon-like peptide 1 (GLP-1) and cholecystokinin (CCK) are secreted by EECs in response to metabolites (5–8, 29). To analyze whether SCFA exposure had an influence on the levels of gut hormone transcripts, STC-1 cells were exposed to propionate and butyrate (5 mM) for 5 h. Interestingly, propionate, but not butyrate, induced a significant decrease in CCK mRNA, while there no significant changes in the levels of GCG mRNA, the gene that encodes preproglucagon (30) (Supplementary Figure 1).

### The Umami Taste Receptor Is Significantly Upregulated by SCFAs, but Not Synthetic FFAR Ligands

**Figure 2:**
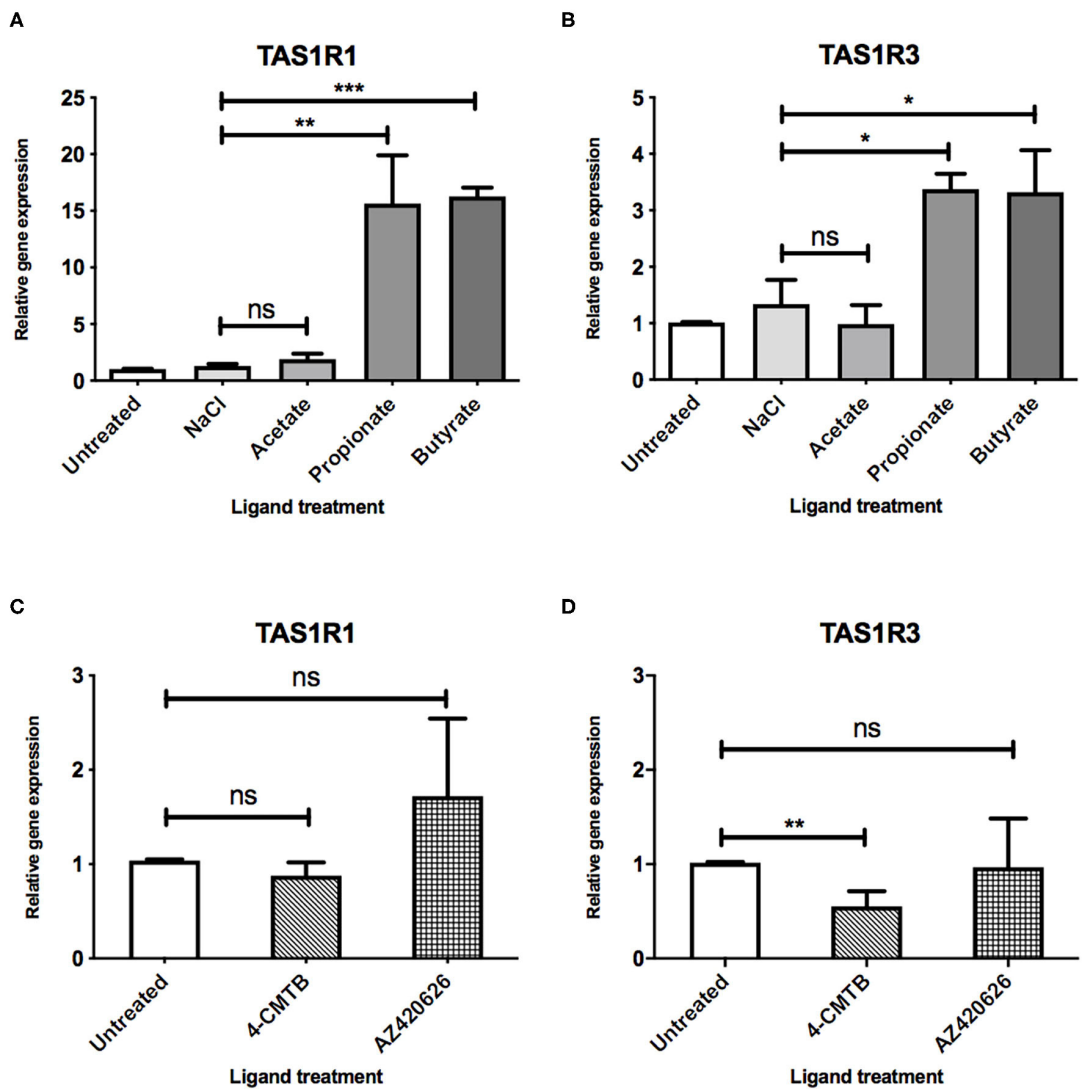
SCFAs and synthetic ligands differ in their ability to upregulate the umami taste receptor. **(A,B)** STC-1 cells were incubated with NaCl or SCFAs (10 mM) for 5 h, after which, RNA was extracted and purified. Expression of taste receptors TAS1R1 **(A)**, TAS1R3 **(B)** was quantified using qPCR analysis and normalized to the levels of housekeeping gene β-actin. Data are expressed as mean ± SEM fold-change in expression over the untreated control (*n* = 3). *T*-tests vs. control; ns, non-significant; *p < 0.05; **p < 0.01; ***p < 0.001. **(C,D)** STC-1 cells were incubated with either 4-CMTB or AZ420626 (10 μM) for 5 h, after which, RNA was extracted and purified. Expression of taste receptors TAS1R1 **(C)**, TAS1R3 **(D)** was quantified using qPCR analysis and normalized to the levels of housekeeping gene β-actin. Data are expressed as mean ± SEM fold change in expression over the untreated control (*n* = 3). Unpaired *t*-tests vs. control; ns, non-significant; *p < 0.05; **p < 0.01; ***p < 0.001.

After demonstrating that a 3:1:1 mixture of SCFAs can influence the expression profiles of components of the umami taste receptor, we assessed whether specific SCFAs mediate these changes. STC-1 cells were treated with either acetate, propionate or butyrate (10 mM) for 5 h, after which, mRNA levels of TAS1R1 and TAS1R3 were measured. Interestingly, incubation with propionate or butyrate, but not acetate, was sufficient to induce significant upregulation of both components of the umami taste receptor. TAS1R1 was upregulated ~15-fold by both propionate (*p* = 0.0186) and butyrate (*p* = 0.0001; Figure 2A). TAS1R3 was upregulated more modestly than TAS1R1, by ~3-fold following propionate (*p* = 0.01) or butyrate (*p* = 0.04) treatment (Figure 2B).

As SCFAs have been reported to be able to activate both FFAR2 and FFAR3 (9), we used synthetic ligands to determine whether selective activation of each receptor had a similar effect on umami taste receptor gene expression. STC-1 cells were exposed to 4-CMTB, a FFAR2-specific agonist, or AR420626, a FFAR3-specific agonist at concentrations known to induce maximal signal responses (12, 14, 15). The ability of these synthetic ligands to activate the Gα_i/o_ signaling, via inhibition of forskolin-induced increases in cAMP levels was also confirmed (See Supplementary Figure 2). While these ligands activate Gα_i/o_ signaling, as do SCFAs, they were not able to upregulate the umami taste receptors (Figures 2C,D). Indeed, 4-CMTB induced a significant 2-fold decrease in mRNA levels of TAS1R3 (Figure 2D).

### SCFA-induced Upregulation of the Umami Taste Receptor mRNA Involves Gα_i/o_

**Figure 3:**
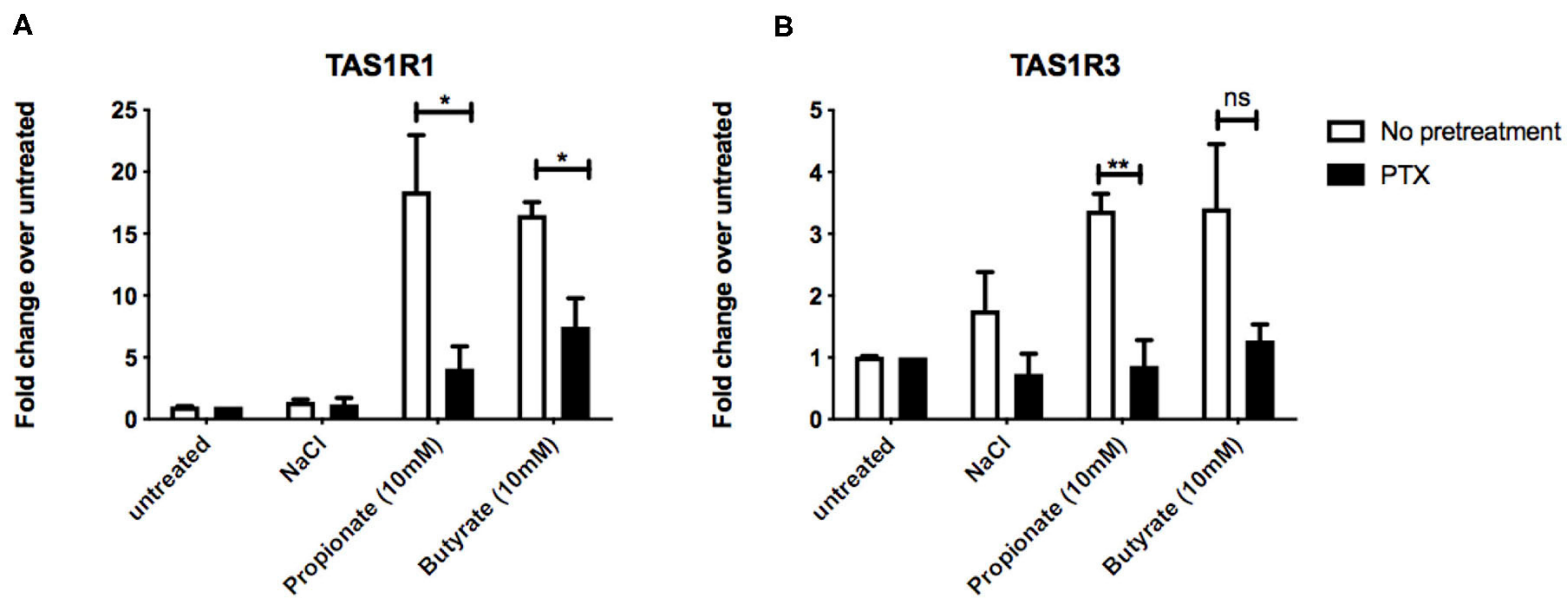
Inhibtion of Gα_i/o_ signaling impacts SCFA-mediated changes in taste receptor gene expression. **(A,B)** STC-1 cells were pretreated with Gα_i/o_ inhibitor pertussis toxin (PTX) (200 ng/μL, 18 h; black bars) or no pretreatment (white bars), followed by stimulation with either NaCl, propionate or butyrate (all 5 mM) for 5 hr. RNA was extracted and purified. Expression of taste receptors TAS1R1 **(A)**, TAS1R3 **(B)** was quantified using qPCR analysis and normalized to the levels of housekeeping gene β-actin. Data are expressed as mean ± SEM fold change in expression over the NaCl control either with or without PTX exposure (*n* = 3). Two-way ANOVA, Bonferroni *post hoc* of no pretreatment vs. PTX treatment for each ligand; ns, non-significant; *p < 0.05; **p < 0.01.

At rodent orthologs of FFAR2 and FFAR3, both propionate and butyrate show significant selectivity for FFAR3 (15) a receptor known to signal via Gα_i/o_ (9). To investigate whether Gα_i/o_ activation plays a fundamental role in the SCFA-induced upregulation of umami taste receptor transcripts, STC-1 cells were incubated for 18 h with pertussis toxin (PTX), a Gα_i/o_ inhibitor. Compared to the basal levels of the PTX-pretreated control, pretreatment of cells with PTX significantly reduced the ability of propionate and butyrate to induce upregulation of TAS1R1, from 18.4- to 4.6-fold for propionate, and from 16.5- to 6.3-fold for butyrate (Figure 3A). PTX-pretreatment completely abolished the propionate- and butyrate-induced upregulation of TAS1R3 (Figure 3B), and inhibited the decrease in CCK transcript observed with propionate incubation (Supplementary Figure 1).

### Umami Taste Ligands Signal in STC-1 Cells in a Manner That Is Potentiated by Addition of IMP

**Figure 4:**
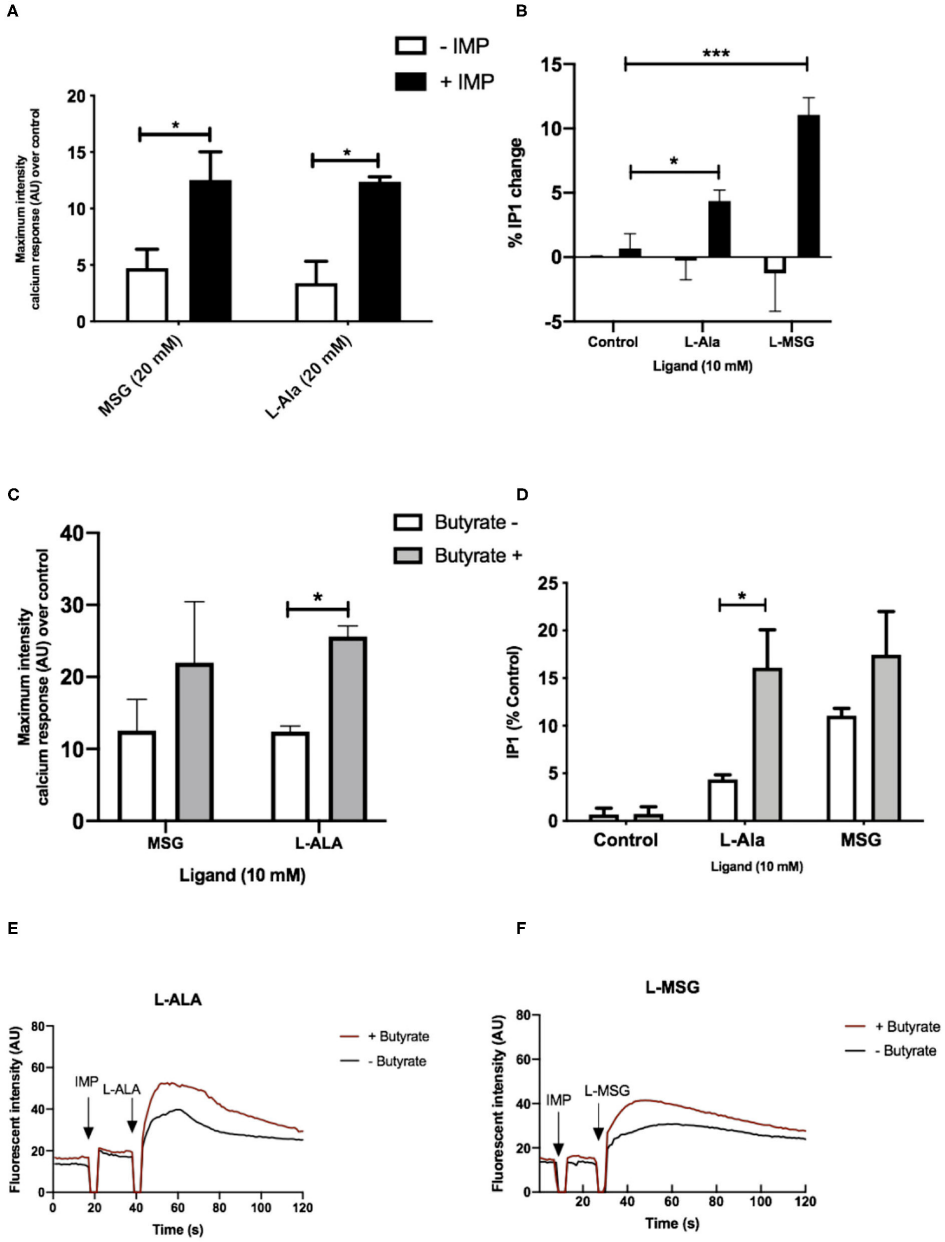
Umami receptor signaling cascades are potentiated by butyrate pretreatment. **(A)** Intracellular Ca^2+^ levels measured in STC-1 cells, incubated with fluorescence Ca^2+^ indicator dye Fluo4-AM following addition of IMP (2 mM, black bars) or NaCl control (white bars; 2 mM) with L-Ala (20 mM) or L-MSG (20 mM). Data is expressed as mean ± SEM maximal fluorescence intensities over the control and is taken from 20 cells per sample, in duplicate (*n* = 3). *T*-test vs. control; *p < 0.05; **p < 0.01; ***p < 0.001. **(B)** Intracellular IP_1_ accumulation measured in STC-1 cells on the addition of IMP (black bars) with either NaCl control (white bars; 2 mM), L-Ala (20 mM), or L-MSG (20 mM). Data is expressed as mean ± SEM across three distinct experiments; *t*-test *p < 0.05; **p < 0.01; ***p < 0.001 vs. control. **(C)** Intracellular Ca^2+^ levels measured in butyrate-naive (white) or butyrate-pretreated (gray) STC-1 cells, incubated with fluorescence Ca^2+^ indicator dye Fluo4-AM, followed by stimulation with IMP (2 mM) with L-Ala (20 mM) or L-MSG (20 mM). Data is expressed as mean ± SEM maximal fluorescence intensities over the control and are taken from 20 cells per sample, in duplicate (*n* = 3). *T*-test vs. control, *p < 0.05; ***p < 0.001. **(D)** Intracellular IP_1_ accumulation measured in butyrate-naive (white) or butyrate-pretreated (gray) STC-1 cells after incubation with IMP (2 mM) and either L-Ala (20 mM) or L-MSG (20 mM). Data is expressed as mean ± SEM (*n* = 3); *t*-test vs. control, *p < 0.05; ***p < 0.001. **(E,F)** Representative fluorescence intensity plots following IMP (2 mM) and L-Ala (**E**; 20 mM) or L-MSG (**F**; 20 mM) stimulation in butyrate pretreated (red lines) and butyrate naive (black lines) STC-1 cells, expressed in arbitrary units (AU).

We then aimed to determine whether the observed upregulation of umami taste receptor mRNA could be translated into an increase in functional umami receptor signaling. The umami taste receptor is sensitive to a number of L-amino acids (L-AA). It is documented that L-Ala elicits the strongest Ca^2+^ signals at the murine umami receptor (27). Thus, we selected L-Ala for use in our assays. We also employed the characteristic umami taste receptor ligand, L-MSG, as it is a substantial component in modern human diets (8–10% of the AA content) (31), and other L-AAs do not activate the human umami receptor to the same extent as L-MSG (27). To confirm the signals were via activation of umami taste receptor, rather than other amino acid-sensitive receptors, we first assessed whether signaling was synergized by IMP, as this is a unique signaling property of the umami receptor (26, 27, 32). Taste receptors have been shown to activate phospholipase C-mediated pathways, leading to formation of 1,4,5-inositol triphosphate (IP_3_) (32), thus, umami taste receptor activation was determined by measurement of intracellular Ca^2+^ and IP_1_, a downstream metabolite of IP_3._ Addition of IMP (2 mM) significantly increased the levels of Ca^2+^and IP_1_ signal induced by both L-MSG and L-Ala (10 mM; Figures 4A,B).

### The Increase in Umami Taste Receptor Transcript on Exposure to SCFAs Is Coupled With an Increase in Signaling Response to Some Umami Taste Ligands

To investigate whether upregulation of TAS1R1 and TAS1R3 mRNA by SCFAs translates into an increase in umami-receptor signaling, we pretreated STC-1 cells overnight with butyrate, at a concentration able to elicit significant upregulation of both transcripts (Figures 2A,B). This treatment time was also chosen to allow for translation, folding, maturation and cell surface targeting (33). We then reassessed the cells' signaling response to L-MSG and L-Ala. Pre-incubation with butyrate significantly increased IP_1_ signaling and the maximum-induced Ca^2+^ response to L-Ala/IMP over time (Figures 4C–F). L-MSG-induced IP_1_ and Ca^2+^ responses exhibited greater variability following butyrate pretreatment than L-Ala responses (Figures 4C–F), potentially because L-MSG is less potent than L-Ala at the rodent umami taste receptor (27). Overall, this data suggests butyrate-induced increases in umami taste receptor mRNA also result in enhanced umami taste receptor activity.

## Discussion

GPCRs expressed in the GI tract have a well-established role in nutrient-sensing and anorectic/incretin gut hormone secretion (3, 5, 7, 8, 16–19). Therefore, developing an understanding of GPCR expression profiles and signaling functions in EECs has therapeutic value in the field of obesity and Type II diabetes. SCFAs modulate gene expression in various cells, tissues and species (19, 34–38); however, this is the first report that physiologically relevant concentrations of SCFAs, particularly propionate and butyrate, can directly and robustly upregulate transcripts encoding GPCRs in EECs. Of particular note was the substantial upregulation of the umami taste receptor subunits, as the expression profile of these is significantly different in the GI tract of obese individuals when compared with lean controls (20, 21). These observations provide a mechanism to explain how diet composition and SCFA production are linked with fluctuations in GPCR expression patterns in obese humans and mice (20–23).

Our work demonstrated that the most highly upregulated taste receptor transcript upon EEC exposure to SCFAs was the umami taste receptor subunit TAS1R1. When both STC-1 cells and murine intestinal organoids were exposed to a mixture of SCFAs at a concentration often found in the colon (4, 17), TAS1R1 was upregulated nearly 7-fold, with no effect on the expression levels of either of the SCFA receptors FFAR2/FFAR3 in STC-1 cells. The umami taste receptor is a known heterodimer of TAS1R1 and TAS1R3 (26, 27, 32). It is co-expressed in GI tissue with CCK (39) and, on activation by protein hydrolysates, induces CCK secretion from EECs (29). Interestingly, exposure to either propionate or butyrate robustly enhanced gene expression of both umami taste receptor subunits, while propionate, but not butyrate, decreased CCK mRNA levels. The decrease in CCK mRNA levels by propionate contradicts prior studies in mouse GLUTag cells and the human NCI-H716 EEC line, in which propionate induced an increase in CCK expression; however, this was after a longer stimulation of 24 h (40), perhaps suggesting that the effects of propionate depend upon whether the exposure is acute vs. chronic. The decrease in CCK levels observed in this study could suggest either decreased gene expression or increased translation of CCK mRNA to protein, the latter being consistent with prior reports of SCFAs inducing enhanced CCK protein content but decreased mRNA levels in a rodent pancreatic islet cell line (41).

There are two plausible mechanisms for the effects of SCFAs on the gene expression of these receptors and gut hormone; via FFAR2/3 G-protein signaling or histone deacetylase (HDAC) inhibition (42). That both propionate and butyrate, but not acetate, can increase the levels of this receptor is interesting, and maybe explained by the difference in potency and affinity of the SCFAs at rodent FFAR2/FFAR3 (15) and at HDACs (42).

Both FFAR2 and FFAR3 couple to Gα_i/o_ signaling, and although FFAR2 is also a known Gα_q/11_-coupled receptor, we have recently demonstrated that while synthetic selective FFAR2 ligands activate Gα_q/11_ signaling in EECs, SCFAs do not (43). Our data in the current study support a role for GPCR-Gα_i/o_ signaling in mediating these changes in gene expression. We clarified the contribution of Gα_i/o_ signaling elicited by SCFAs (9, 12, 14, 15) by inhibiting FFAR2/3 Gα_i/o_ signaling with PTX, which significantly reversed the changes in both umami taste receptor subunits and CCK mRNA that were induced by either propionate or butyrate. This suggests FFAR2/3 Gα_i/o_ signaling contributes significantly to the upregulation, even for butyrate; a very potent HDAC inhibitor (<1 mM) (42). Inhibition of Gα_i/o_ activity abolished SCFA-induced TAS1R3 upregulation, and significantly reduced propionate-induced TAS1R1 upregulation more than butyrate-induced upregulation. This, together with the knowledge that propionate cannot inhibit HDACs as potently as butyrate [only doing so at high concentrations of >10 mM (42)], suggests that propionate may act predominantly via a FFAR2/3 signaling mechanism, while butyrate may act through both receptor signaling and HDAC inhibition. If propionate is acting via FFAR2/3 to modulate gene expression, it may be hypothesized that FFAR3 is the more likely candidate, as propionate is nearly ten times more selective for rodent FFAR3 than FFAR2 (15). Furthermore, FFAR3 signaling influences gene expression in other cellular models: FFAR3 knock-out murine pancreatic islets have significantly different transcriptomes to wild-type animals, though in genes associated with insulin secretion and glucose regulation (44).

Surprisingly, synthetic FFAR2/FFAR3 selective ligands could not upregulate TAS1R1 or TAS1R3 transcripts, despite their ability to activate upstream receptor signaling in EECs. This is consistent with our recent findings that endogenous SCFA and synthetic ligands have distinct activation profiles at SCFA receptors (43), and thus, potentially elicit different downstream responses. If both SCFAs and synthetic ligands activate similar upstream G-protein pathways, it remains to be determined the additional mechanisms that drive the SCFA-selective increases in gene transcription of umami taste receptors, but potentially suggests a role for ligand-induced bias signaling at FFAR2/3.

We then determined whether SCFAs could enhance functional umami taste receptor activity in STC-1 cells. Other GPCRs able to sense L-AAs are also expressed in EECs (8, 45), and there were some technical challenges in deciphering the precise contributions of each L-AA-sensitive GPCR, owing to the lack of selective ligands. However, the synergistic effects of IMP offered a mechanism to detect umami-specific responses (26, 27). Here, umami ligands, L-MSG and L-Ala, only induced increases in Ca^2+^and IP_1_ in the presence of IMP, as observed when TAS1R1-TAS1R3 is expressed in other heterologous systems (26, 27), supporting a role for TAS1R1-TAS1R3 signaling in STC-1 cells. Our data demonstrate a significant increase in umami taste receptor signal activity after pretreatment with butyrate. Of course, we cannot rule out that butyrate may modulate the expression of other genes involved in Ca^2+^ signaling, such as Ca^2+^ channels or other L-AA-sensitive GPCRs (8, 45–47), but it is still interesting to consider that butyrate exposure enhances L-Ala/IMP-induced Ca^2+^ signaling. This is a classical pathway associated with secretion of anorexergic gut hormones in EECs, which, in turn, elicit positive physiological effects, including blood glucose regulation and appetite reduction. Although butyrate alone does not induce gut hormone secretion, under conditions where a mixture of SCFAs are present, it may augment responses from other metabolites, including propionate. Thus, it will be interesting in future to see if there are alterations in taste receptor activity by propionate exposure (1, 5, 7, 8, 16, 19, 45).

Our study is not without its limitations. The utilization of STC-1 cells, an immortalized EEC cell-line, means that results may not be representative of the behavior of EECs *in vivo* (24). However, we did observe that SCFAs induced a robust increase in umami taste receptor gene expression in small intestinal organoids; a 3D *in vitro* model we have previously employed to study L-cell function (48). We also need to consider translation of the study to human systems and whether SCFAs would alter similar or distinct gene expression profiles. Although transcriptomic analysis of human EEC populations have demonstrated that the expression profile correlates strongly with murine counterparts, there are some discrepancies in GPCR transcripts, which may have the potential to impact results (49). The signaling properties of GPCRs are also not completely conserved from species to species, with regard to both ligand affinity and downstream signaling cascades (15, 27). Therefore, any conclusion we make in mouse models may not necessarily be consistent with other species, although *in vivo* studies of SCFAs in regulating appetite and metabolism are consistent between mouse models and humans (5, 16, 18). Interestingly, studies have demonstrated that diet supplementation with L-AAs can directly regulate transcript levels of TAS1R1 and TAS1R3 in porcine jejunum tissue, via a mechanism dependent on umami taste receptor signaling, which led to CCK secretion (50). Future studies will endeavor to address the translation of these findings to humans, and whether SCFAs can directly alter responsiveness of the gut to a specific or broad set of metabolites/dietary molecules that regulate gut hormone release.

The temporal nature of these changes, in terms of kinetics and the duration of their persistence *in vivo*, will also be important future steps to translate these findings. We must consider the entire EEC environment—the spatial and temporal exposure to multiple, fluctuating GPCR ligands (both luminal and paracrine/autocrine)—and how this may regulate protein expression and signaling pathways, alongside other molecular mechanisms, such as GPCR ligand bias, spatial-directed signaling and heteromerization; all of which further diversify this complex signaling network. This will indefinitely be a challenge, owing to the indiscriminate nature of factors that may contribute to GPCR expression fluctuations throughout the body, including metabolic status, gut microbiota composition and diet constitution, which may oppose or potentiate the signaling of SCFAs.

SCFAs induce gut hormone secretion via signaling through their GPCRs (12, 16, 18). Using the evidence gathered here, it is highly plausible that SCFAs also act to “reprogram” EECs to distinct, seemingly unrelated, dietary nutrients, by upregulating the counterparts receptive to their signaling. Despite limitations, our findings support the concept that GPCR signaling networks in EECs are highly complex, exhibiting the potential to adapt in response to dynamic fluctuations of bioactive nutrients (8, 18–23, 36, 45, 51, 52). In summary, we can conclude that SCFA-induced remodeling of the GPCR signal system is an interesting and novel area that needs to be explored further, as it has potential therapeutic value.

## Data Availability Statement

The raw data supporting the conclusions of this article will be made available by the authors, without undue reservation.

## Author Contributions

MS performed all experiments in STC-1 cells under supervision of ET, GF, and AH. YM cultured and carried out experiments in intestinal organoids. MS, AB, YM, ET, GF, and AH designed research and analyzed data and wrote the paper. All authors contributed to the article and approved the submitted version.

## Conflict of Interest

AB is a shareholder in Heptares Therapeutics (part of the Sosei Group) and hold stock options in the Sosei Group. The remaining authors declare that the research was conducted in the absence of any commercial or financial relationships that could be construed as a potential conflict of interest.
