# Supplementary material for: Short Chain Fatty Acids Enhance Expression and Activity of the Umami Taste Receptor in Enteroendocrine Cells via a Gαi/o Pathway
Source: Front Nutr. 2020 Oct 29;7:568991. doi: 10.3389/fnut.2020.568991 (PMC7658341; doi:10.3389/fnut.2020.568991)
Supplement: Supplementary file 1 [file Data_Sheet_1.PDF]

Supplementary Material

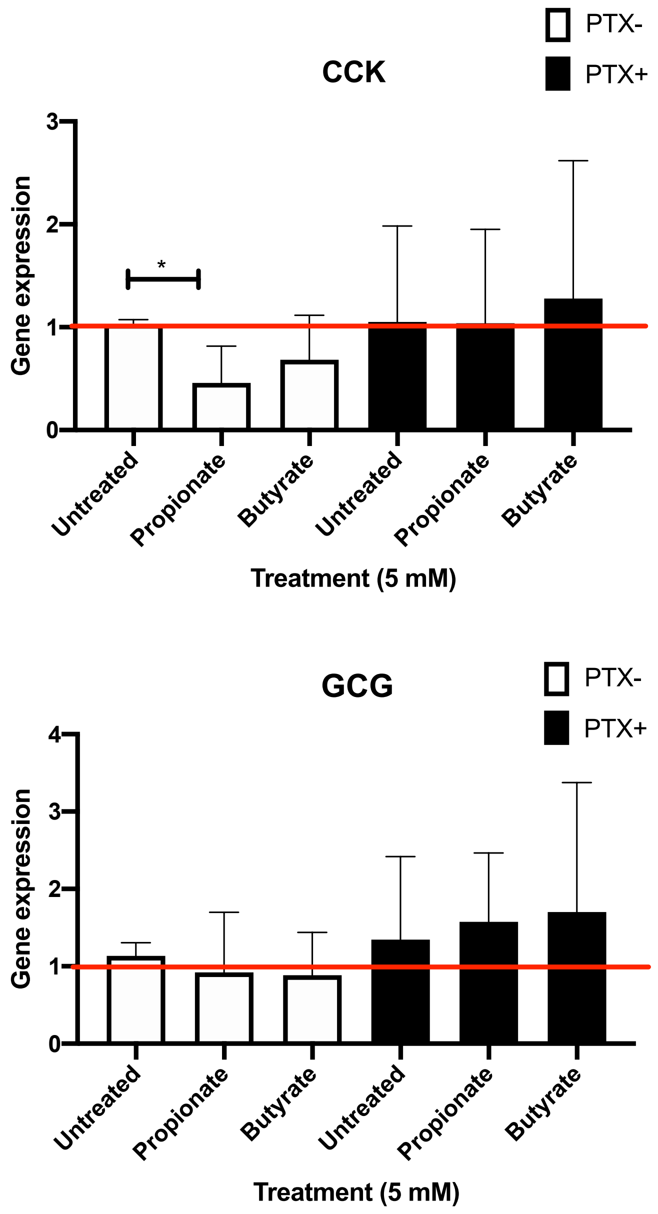

Supplementary Figure 1. SCFA-mediated changes in CCK gene expression is via a G$\alpha_{i/o}$-dependent pathway. A-B) STC-1 cells were pretreated with G$\alpha_{i/o}$ inhibitor pertussis toxin (PTX) (200 ng/µL, 18 hrs; black bars) or no pretreatment (white bars), followed by stimulation with either NaCl (untreated), sodium propionate or sodium butyrate (all 5 mM) for 5 h. RNA was extracted and purified. Expression of CCK or GCG (preproglucagon) was quantified using qPCR analysis and normalized to the levels of housekeeping gene β-actin. Data are expressed as mean ± SEM fold change in expression over the NaCl control either with or without PTX exposure (n=3). Two-way ANOVA, Bonferroni post hoc of no pretreatment vs. PTX treatment for each ligand; *$p<0.05$;

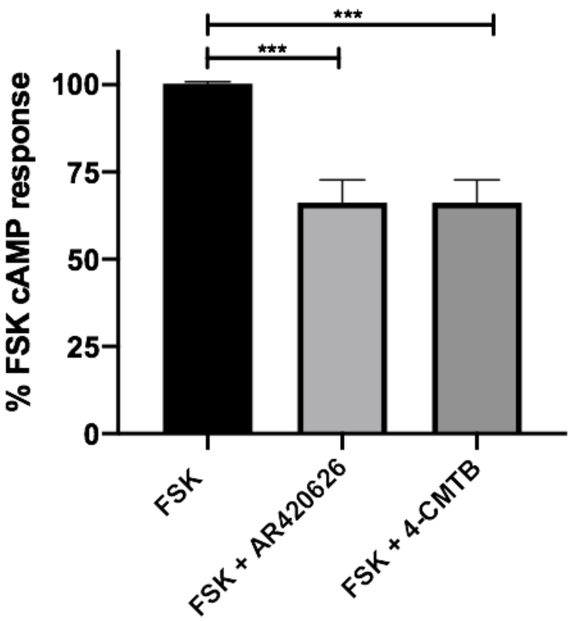

Supplementary Figure 2. Selective FFAR2 and FFAR3 ligands activate Gαi signaling. Intracellular cAMP accumulation measured in STC-1 cells on the addition of forksolin (3 µM) alone or with the addition of FFAR3-specific agonist AR420626 (10 µM) or FFAR2-specific agonist 4-CMTB (10 µM). Data is expressed as the percentage cAMP concentration compared to the FSK-only control (mean ± SEM; n=3); t-test *p<0.05; **p<0.01; ***p<0.001 vs. FSK-only control.

1.1 Supplementary Tables

Gene	Forward (5'-3')	Reverse (5'-3')
**α-gustducin**	TAGGAGCCGAGAGGACCAAG	GCTGGTATTCAGATGCCCTTTC
**β-actin**	GGCTGTATTCCCCTCCATCG	CCAGTTGGTAACAATGCCATGT
**FFAR2**	CGAGAACTTCACCCAAGAGC	TGAGGGAACTGAACACCACA
**FFAR3**	CAATACTCTGCATCTGTGAC	CAGGTAGACGGAAAAGAAA
**TAS1R1**	TGGAGGAGTGGTTGCGAAGA	TCCATGCCAACGTGAAGAGC
**TAS1R2**	TCCATGCCAACGTGAAGAGC	GCTACAGTTGTTGATTTCCTCCA
**TAS1R3**	TGCTGCCTACTGCAACTACAC	CCGGTCACTTAGCCGATCC
**TAS2R(108)**	TGACACGTCATTTGACCTCAG	GCTGGTCCTGTTTCTCTGCAT
**CCK**	AGCGCGATACATCCAGCAG	ACGATGGGTATTCGTAGTCCTC
**GCG**	TTACTTTGTGGCTGGATTGCTT	ATGGGCGTTTGTCTTCATTCA

Supplementary Table 1.

The forward and reverse primer sequences used for quantitative polymerase chain reaction (qPCR) analysis of the murine isoforms of house-keeping gene β-actin and the nutrient sensing receptors FFAR2, FFAR3, TAS1R1, TAS1R2, TAS1R3, TAS2R(108), TAS2R(138), the gustatory G-protein α-gustducin and the gut hormones CCK (cholecystokinin) and GCG (preproglucagon).
